# Phylogeny, evolution, and potential ecological relationship of cytochrome CYP52 enzymes in Saccharomycetales yeasts

**DOI:** 10.1038/s41598-020-67200-5

**Published:** 2020-06-24

**Authors:** Jossue Ortiz-Álvarez, Arturo Becerra-Bracho, Alfonso Méndez-Tenorio, Jazmin Murcia-Garzón, Lourdes Villa-Tanaca, César Hernández-Rodríguez

**Affiliations:** 1 Laboratorio de Biología Molecular de Bacterias y Levaduras. Departamento de Microbiología, Escuela Nacional de Ciencias Biológicas, Instituto Politécnico Nacional, Prol. de Carpio y Plan de Ayala s/n. Col. Sto. Tomás, 11340 Ciudad de México, México; 2 Facultad de Ciencias, Universidad Nacional Autónoma de México, Apdo. Postal 70–407, Cd. Universitaria, 04510 Ciudad de México, México; 3 Departamento de Bioquímica, Escuela Nacional de Ciencias Biológicas, Instituto Politécnico Nacional, Prol. de Carpio y Plan de Ayala s/n. Col. Sto. Tomás, 11340 Ciudad de México, México; 4 Present Address: Centro de Biotecnología Genómica, Instituto Politécnico Nacional. Blvd. del Maestro S/N Esq. Elías Piña. Col. Narciso Mendoza, 88710 Reynosa, Tamaulipas México

**Keywords:** Molecular evolution, Protein function predictions, Molecular ecology, Computational biology and bioinformatics, Ecology, Evolution, Microbiology, Fungi

## Abstract

Cytochrome P450s from the CYP52 family participate in the assimilation of alkanes and fatty acids in fungi. In this work, the evolutionary history of a set of orthologous and paralogous CYP52 proteins from Saccharomycetales yeasts was inferred. Further, the phenotypic assimilation profiles were related with the distribution of cytochrome CYP52 members among species. The maximum likelihood phylogeny of CYP52 inferred proteins reveled a frequent ancient and modern duplication and loss events that generated orthologous and paralogous groups. Phylogeny and assimilation profiles of alkanes and fatty acids showed a family expansion in yeast isolated from hydrophobic-rich environments. Docking analysis of deduced ancient CYP52 proteins suggests that the most ancient function was the oxidation of C4-C11 alkanes, while the oxidation of >10 carbon alkanes and fatty acids is a derived character. The ancient CYP52 paralogs displayed partial specialization and promiscuous interaction with hydrophobic substrates. Additionally, functional optimization was not evident. Changes in the interaction of ancient CYP52 with different alkanes and fatty acids could be associated with modifications in spatial orientations of the amino acid residues that comprise the active site. The extended family of CYP52 proteins is likely evolving toward functional specialization, and certain redundancy for substrates is being maintained.

## Introduction

Cytochromes P450 (CYPs) are an extended heme-thiolate enzyme superfamily that are widely distributed among different biological domains^1,2^. P450s are involved in the oxidation of myriad endogenous and xenobiotic hydrophobic compounds. Hence, P450s play a critical role in the biosynthesis of structural molecules and secondary metabolites^3,4^, utilization of compounds as sole carbon and energy sources^5^, and cellular detoxification^6^, among others.

The cytochrome P450s that belong to the CYP52 family are present in the orders Eurotiales, Pezizomycetes, Leotiomycetes, Dothideomycetes and Saccharomycetales of Ascomycota fungi^7^. CYP52 enzymes are located in the endoplasmic reticulum (ER) membrane, and their main function is the hydroxylation of *n*-alkanes and fatty acids, which are successively oxidized to mono- or dicarboxylic fatty acids, respectively, by additional oxidation reactions catalyzed by alcohol and aldehyde deshydrogenases^5,8^. Finally, fatty acids are degraded in the fungal peroxisome and mitochondria via the β-oxidation pathway to CO_2_. This degradation produces acetyl-CoA, FADH_2_, and NADH^9–12^.

*Candida albicans*, *Candida maltosa*, *Candida tropicalis*, and *Yarrowia lipolytica* are model Saccharomycetales used for the study of function and transcriptional regulation of multiple orthologous and paralogous P450 encoded by CYP52 genes^7,13–18^. The CYP52 genes in *C. maltosa, Candida pseudoglaebosa, C. tropicalis*, *Kodamaea ohmeri*, and *Y. lipolytica* are induced by *n*-alkane or fatty acid substrates^13,14,16,19^ and repressed by glycerol^13,16^ or glucose^20^.

*Y. lipolytica* harbors a broad collection of CYP52 enzymes encoded by *ALK* genes^8^. Deletion of *ALK* genes in *Y. lipolytica* demonstrated that *ALK1* and *ALK10* are involved in the assimilation of C10-C18 *n*-alkanes, whereas *ALK2* and *ALK9* prefer C15-C18 *n*-alkanes. The *ALK4*, *ALK5*, and *ALK7* genes participate in the assimilation of dodecanoic acid, and the *ALK3* and *ALK6* genes are involved in the assimilation of both long-chain *n*-alkanes and dodecanoic acid^18^. In brief, CYP52 paralogous enzymes of *Y. lipolytica* are multifunctional, partially redundant, and exhibit a limited specificity for substrate. Furthermore, there is an apparent transcriptional redundancy of CYP52 genes with different substrates, as shown by reverse transcription polymerase chain reaction (RT-PCR) and Northern blot analysis^16,18^.

In Saccharomycetales yeast, selective pressures drive the maintenance of a smaller genome size compared to other fungi^21,22^ and a moderate/high genomic content of coding genes between 55 and 70%^23^. However, many fungi maintain some multigene families, which can comprise approximately 30–45% of the genome^24,25^. A very expanded family of CYP52 orthologous and paralogous enzymes are harbored among yeast^25^. In particular, the number of paralogs of several protein families in Saccharomycetales is variable: it ranges from two to 19 copies^24,26^. The paralogous proteins can present partial or total redundancy for their biochemical activity or expression profile or can be functionally independent^27,28^. The redundancy can contribute to robustness because paralogs present fully overlapping functions or can display small differences in their expression or enzyme-substrate interaction. These differences can compensate for a loss the function in the case of genetic alterations or mutations of one of the paralogs^29^. However, the paralogous proteins also can impart fragility when there is an evident functional dependence among them because the deletion of a duplicate can notably affect the function of their pair^30^. Both phenomena can co-exist in different genes and confer adaptation of the yeasts to environmental perturbations^31^.

In this work, the phylogeny, *in silico* reconstruction of three-dimensional structure and enzyme-substrate interaction, and comparison of primary sequence and folding differences between ancient and modern CYP52 were used to predict the hypothetical evolutionary events and possible ecological implications of the extensive orthologous and paralogous CYP52 families of Saccharomycetales yeast.

## Results

### Phylogeny and distribution of the CYP52 family

Deduced CYP52 amino acid sequences were detected and collected from genome yeast projects of Debaryomycetaceae (CTG clade) and Dipodascaceae families. Most of the analyzed genome projects harbored three to seven CYP52 paralogous proteins by haploid genome, with the exception of *Y. lipolytica*, which harbored 12 CYP52 paralogous (Table 1).

**Table 1:** Distribution of orthologous and paralogous CYP52 protein sequences in 13 Saccharomycetales genomes.

Organism	Entry sequence	Source
*Candida albicans*	XP705076, XP713577, XP712906, XP715804	NCBI
*Candida dubliniensis*	CD3671370, XP002421081, XP002421583, XP002421582, XM002422177	NCBI, KEGG
*Candida tropicalis*	CTRG03114, CTRG04959, CTRG02725, CTRG01060, CTRG01061, CTRG03115, CTRG03120	KEGG
*Candida maltosa*	D00481, X55881, X55882, D12716, D12717, Q15588, Q15589	NCBI
*Candida parapsilosis*	CPAR2600870, CPAR2203780, CPAR2800510, CPAR2800520, HE605207, CPAR2204210, CPAR2204220,	NCBI, KEGG
*Candida orthopsilosis*	CORT0A06350, CORT0H01020, CORT0F01930, CORT0D03890,	KEGG
*Lodderomyces elongisporus*	XP001525331, XP001527474, XP001525527, XP001525528, LELG05768	NCBI, KEGG
*Debaryomyces hansenii*	XP4577727, XP457792, XP460110, XP460111, XP460112	NCBI
*Meyerozyma guilliermondii*	PGUG01238, PGUG05670, PGUG05855, PGUG04005	KEGG
*Spathaspora passalidarum*	SPAPADRAFT67265, SPAPADRAFT153278, SPAPADRAFT59378, XP007374114	NCBI, KEGG
*Scheffersomyces stipitis*	XP001383710, XP001383506, XP001383636	NCBI
*Candida tenuis*	CANTEDRAFT113909, CANTEDRAFT116673, CANTEDRAFT120218, CANTEDRAFT130130	KEGG
*Clavispora lusitaniae*	CLUG04851, CLUG03984, CLUG04098	KEGG
*Yarrowia lipolytica*	AB010388, AB010397, AB010389, XP501667, AB010393, AB010392, AB010394, AB010390, AB010391, AB010395, AB010396, AB010399	NCBI

**Figure 1:**
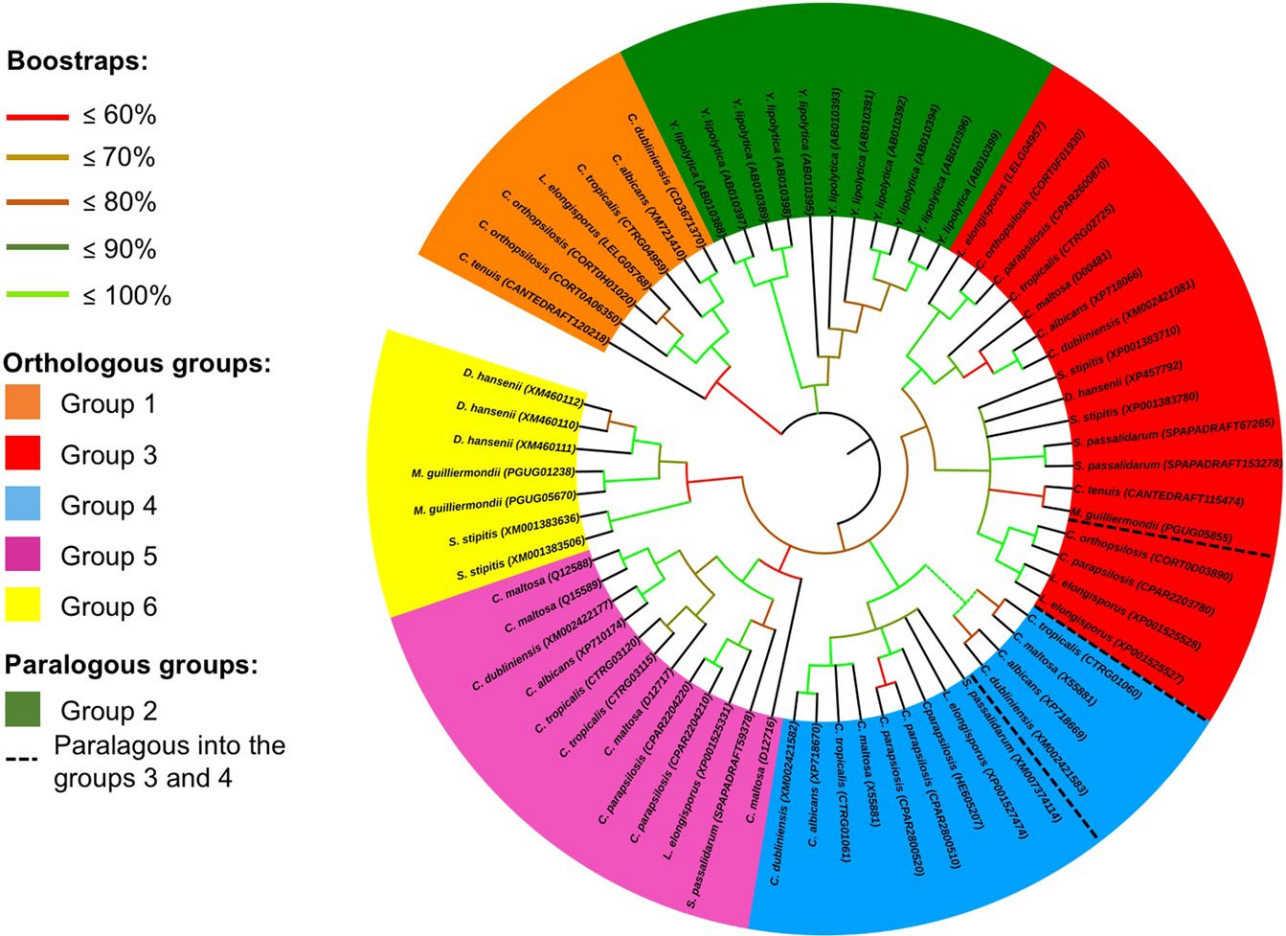
Collapsed maximum likelihood phylogenetic tree constructed with CYP52 amino acid sequences. The WAG + G + I + F evolutionary test was used for phylogenetic reconstruction. The numbers at the nodes represent the Bootstrap values performed with 1000 replicates. Branch lengths are proportional to the number of substitutions per site (see scale bar).

**Figure 2:**
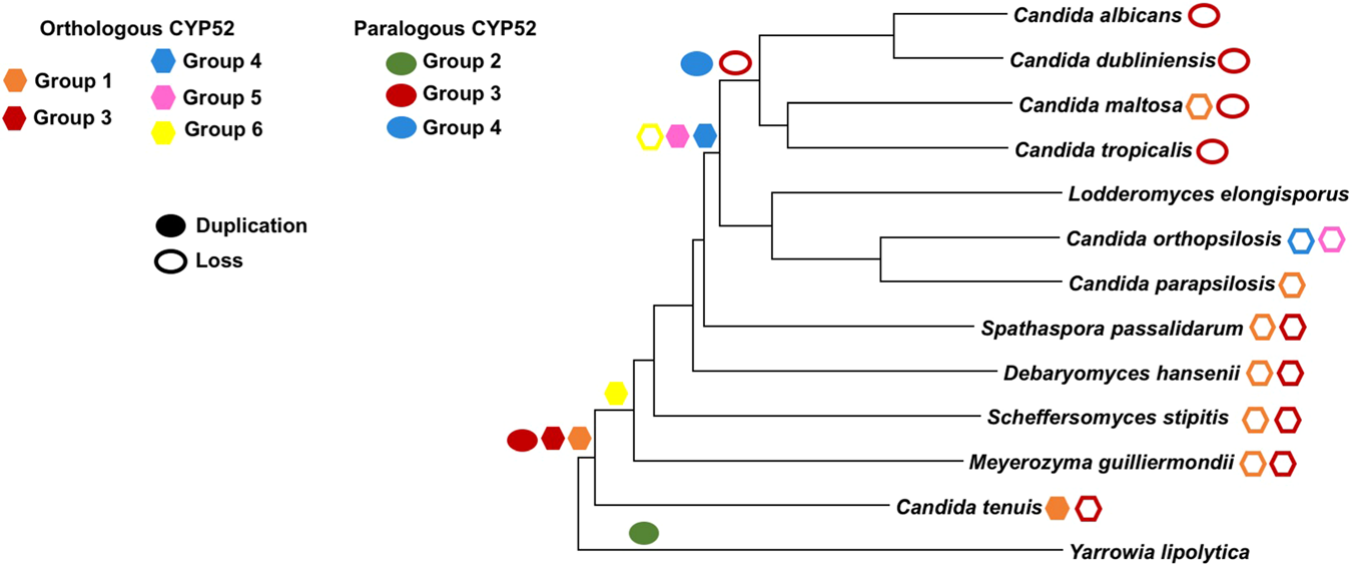
Duplication and loss events in the evolution of orthologous and paralogous CYP52 enzymes. The prediction was performed based on the phylogenomic relationship of the Saccharomycetales yeast tested in this study. The phylogenomic tree was constructed using with a virtual hybridization technique (VGF). Colored forms represent duplication events and non-colored forms represent loss events.

Amino acid sequences were clustered in six orthologous/paralogous groups (Fig. 1). The topology of each CYP52 orthologous groups was consistent with the phylogenomic tree of Saccharomycetales (Fig. 2). A set of 10 non-grouped CYP52 sequences with bootstrap values <0.5 were not clustered into the main orthologous groups and was not used for further analyses. Groups 1, 5, and 6 were defined by at least one orthologous protein per species. Groups 3 and 4 included two closely related orthologous groups; each of them represents paralogous pairs between them. *Y. lipolytica* was clustered in paralogous group 2. Each orthologous, orthologous/paralogous, and exclusively paralogous group clustered between one and 12 paralogous CYP52 proteins per species. However, most of the yeast presented two or more recent paralogous CYP52 members clustered within the same orthologous group (for example, *Candida parapsilosis* in group 5 and *Debaryomyces hansenii* in group 6, among others). In group 1, only one orthologous protein per species was observed, except for *Candida orthopsilosis*, which harbored two paralogs (Fig. 1). Group 1 orthologous CYP52 members maintained a moderate similarity/identity value of approximately 55.9–99.2/33.4–95.3% (Table S1). All the *Y. lipolytica* CYP52 members were clustered in the exclusively paralogous group 2, which maintained relatively low internal similarity/identity values between 55.2–90.4/36.7–68.1% (Table S1). Group 3 and 4 displayed two closely related orthologous proteins. In group 3, a second duplication event occurred for the ancestor to the *C. parapsilosis*, *C. orthopsilosis*, and *Lodderomyces elongisporus* group to yield a second orthologous clade. A third recent event was observed exclusively in *L. elongisporus*. All proteins in group 3 reached a similarity/identity value of 68.3–98.5/53.5–94.9% (Table S1). Two paralogous families of group 4 included six species with similarity/identity values between 74.3–97.9/58.7–93.9% (Table S1). Similar to group 3, recent *C. parapsilosis* duplication events occurred. In group 5, *C. maltosa* displayed three duplication events that generated four paralogous proteins (Fig. 1). Besides, *C. tropicalis* and *C. parapsilosis* exhibited a pair of recent paralogs. Group 6 is a small group formed by only three species (Fig. 1), in which several duplication events can be deduced in all species that generate paralogous groups that are highly related, with similarity/identity values between 73.6–90.6/57.8–81.5% (Table S1).

### Phylogenomic relationship among Saccharomycetales yeast and their correlation with CYP52 family expansion

The whole genome analysis via the VAMPhyRE method revealed that *Y. lipolytica* was the most distant relative compared to the other analyzed yeasts. This finding confirmed its position as the root of the tree (Fig. 2). The tree also showed the close relationship between *C. albicans*, *Candida dubliniensis*, *C. maltosa*, and *C. tropicalis*, whereas *C. orthopsilosis*, and *C. parapsilosis* clustered together with *L. elongisporus*. Despite these findings, they all share a common ancestor. Furthermore, *D. hansenii*, *Candida tenuis*, *Meyerozyma guilliermondii*, *Spathaspora passalidarum*, and *Scheffersomyces stipitis* were segregated into independent groups. Phylogenomics was used to infer the duplication and loss events of CYP52 orthologs and paralogs along the evolution of the studied yeast (Fig. 2). Additionally, two ancient CYP52 losses occurred during evolution for groups 3 and 6. These events involved the loss of the CYP52 of group 6 in the ancestor of all *S. passalidarum* and *Candida/Lodderomyces* clades (Fig. 2). In group 3, the CYP52 loss event apparently involved *S. passalidarum*, *D. hansenii*, *S. stipitis*, *M. guilliermondii*, *and C. tenuis* (Fig. 2). However, a paralogous CYP52 loss event for group 3 occurred in *C. albicans*, *C. dubliniensis*, *C. maltosa*, and *C. tropicalis*. A recent loss event occurred in group 1 involving to *C. maltosa*, *C. parapsilosis*, *S. passalidarum*, *D. hansenii*, *S. stipitis*, and *M. guilliermondii*. Finally, two additional loss events in groups 4 and 5 were observed in *C. orthopsilosis* (Fig. 2).

### Genotypic analysis of *C. tropicalis* strains

The evaluated random amplified polymorphic DNA (RAPD) primers produced a total of four polymorphic and three monomorphic bands (Fig. S1A). The dendrogram generated from the presence/absence matrix showed that environmental strains were different compared to clinical strains. Particularly, *C. tropicalis* 2409, tested in this work, showed a similarity coefficient of 100% in comparison with the other environmental strains. The similarity coefficient ranged from 75–100%; these data revealed moderate diversity among strains (Fig. S1B).

### Alkanes and fatty acid assimilation profiles

**Figure 3:**
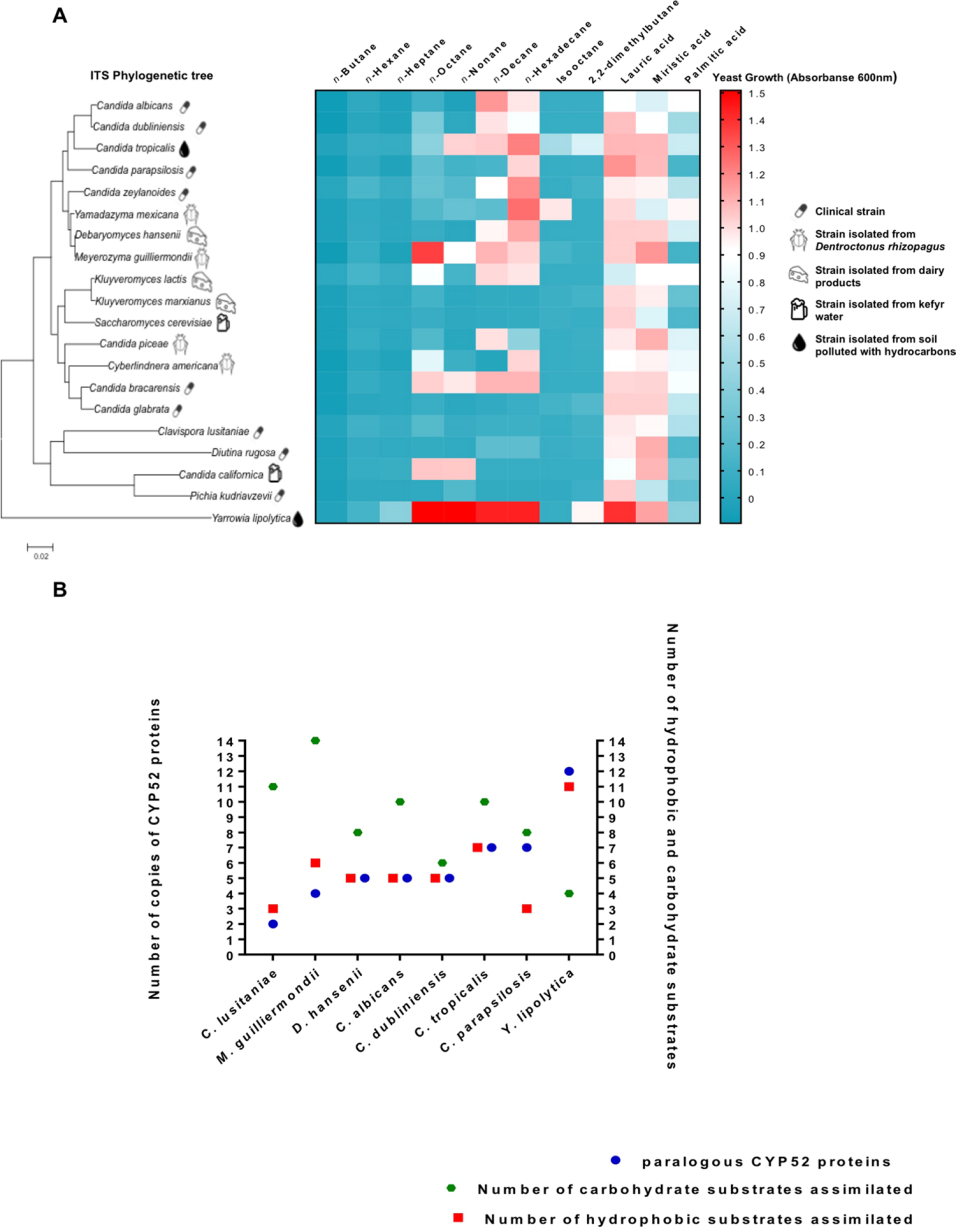
Hydrophobic substrate assimilation profiles and their correlation with the number of CYP52 orthologous and paralogous harbored in Saccharomycetales. (**A**) Growth profiles at 216 h of incubation with various alkanes and fatty acids as the sole carbon sources (P < 0.001; alpha value = 0.05, Two‐way ANOVA analysis, and Tukey’s honestly significant difference post hoc test). (**B**) Correlation between the number of carbohydrate and hydrophobic substrates with the number of CYP52 copies in the Saccharomycetales genomes.

All yeast species assimilated fatty acids (n = 22), but only 17 species were capable of assimilating various *n*-alkanes (Fig. 3A; P < 0.001). Most of the yeasts presented a significant preference for growing with *n*-decane and *n*-hexadecane as the sole carbon sources but exhibited poor growth with short-chain *n*-alkanes (C4-C10) (Fig. 3A). *C. tropicalis*, *M. guilliermondii*, *Candida bracarensis*, and *Y. lipolytica* displayed the widest profile of *n*-alkane assimilation among yeast. No CYP52 enzymes were detected in the WGD clade. However *Kluyveromyces lactis*, *Candida piceae*, *Cyberlindnera americana*, and *C. bracarensis* were able to assimilate C8-C16 *n*-alkanes. Pairs of phylogenetically related species, such as *K. lactis/Kluyveromyces marxianus* and *C. bracarensis/Candida glabrata*, exhibited radically different behaviors in terms of *n*-alkane (C8-C16) assimilation. *C. glabrata*, *K. marxianus*, and *Saccharomyces cerevisiae* were unable to assimilate any type of hydrocarbon as a sole carbon source, although they assimilated long chain fatty acids. In general, the yeasts were unable to use branched alkanes, even though *K. lactis*, *Y. lipolytica*, and *Yamadazyma mexicana* grew poorly with these substrates. Yeast growth with C16 fatty acids was lower compared to C12-C14 fatty acids; some species, including *C. parapsilosis*, *M. guilliermondii*, *K. marxianus*, and *S. cerevisiae*, were unable of growth with palmitic acid.

In general, the yeast species with a low number of CYP52 copies assimilated a limited number of fatty acid and *n*-alkane substrates, whereas yeast species whose genomes harbored an expanded CYP52 protein family assimilated an extended profile of hydrophobic substrates as a sole carbon source (Fig. 3B; Table S2). In contrast, the number of assimilated carbohydrates revealed a tendency contrary to the number of CYP52 copies and number of assimilated hydrophobic substrates (Fig. 3B; Table S2).

### Enzyme-substrate interaction of ancient CYP52 enzymes (anc-CYP52)

**Figure 4:**
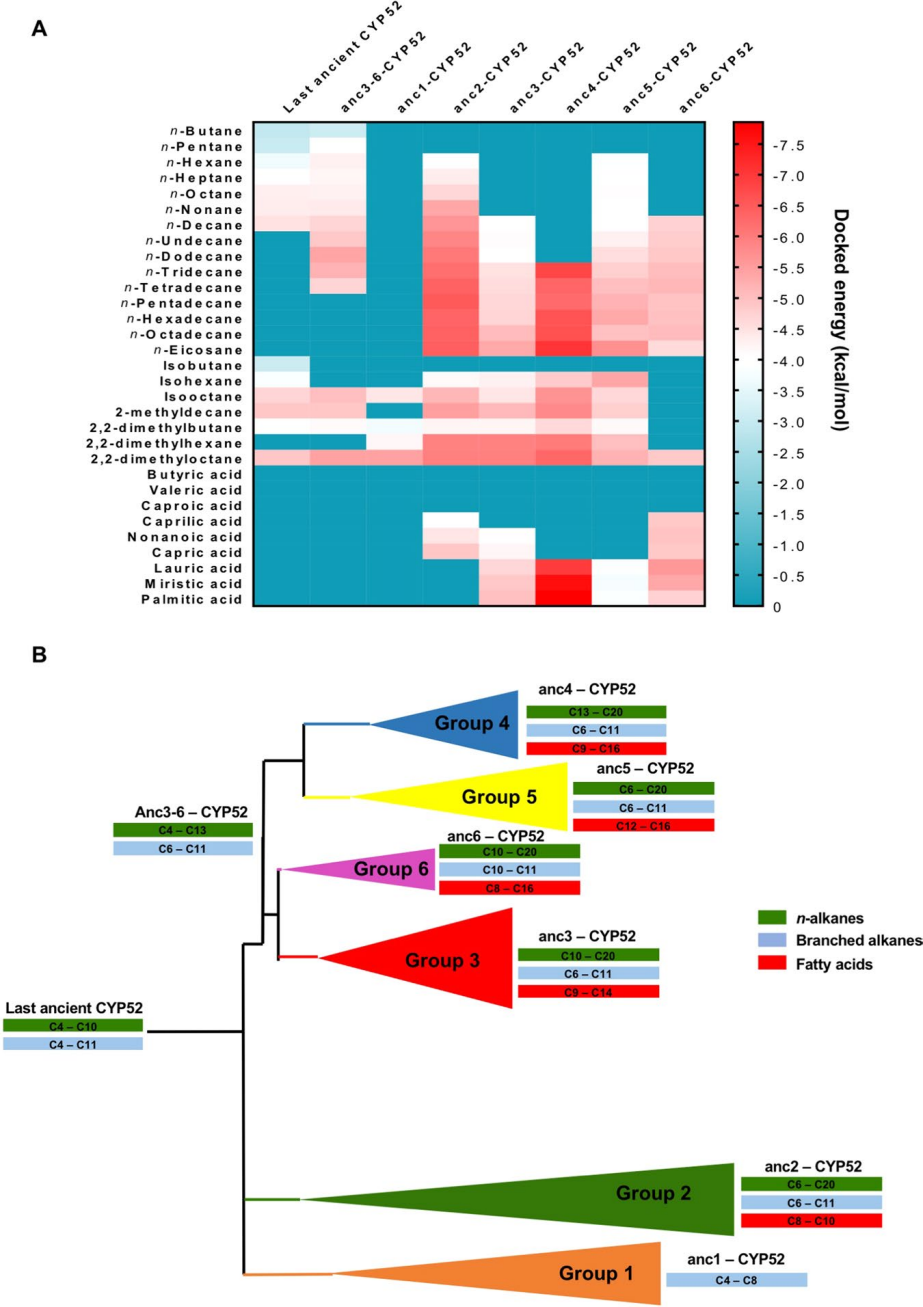
Enzyme-substrate interaction of ancient CYP52 enzymes (anc-CYP52). (**A**) Docked value profiles of the alkane and fatty acid ligands with ancient CYP52 enzymes. (**B**) Evolution of the interaction spectrum into anc-CYP52. The interaction spectrum profiles are indicated in green, blue, and red boxes.

A total of eight ancient CYP52 three-dimensional structures that corresponded to the last ancient CYP52 (last ancient-CYP52), common ancient of groups 3, 4, 5 and 6 (anc3–6-CYP52), ancient CYP52 of each group (anc1-CYP52, anc3-CYP52, anc4-CYP52, anc5-CYP52, and anc6-CYP52), and the ancient CYP52 for the *Y. lipolytica* paralog (anc2-CYP52) were modelled. The results of ancestral reconstruction using PAML package are summarized in Table S3. The ancient CYP52 three-dimensional structures maintained an equilibrium system during the simulation at 2,000,000 steps during 1 ns (Fig. S2). For the purposes of this work, docked energy values were considered moderate when interactions among the CYP52 catalytic site and ligands had values between −2.5 and −5.5 kcal/mol, and high when values were between −5.6 and −7.5 kcal/mol (Fig. 4A). The last ancient CYP52 structures displayed moderate docked energy values with C4-C10 *n*-alkanes and branched alkanes (Fig. 4A). There were no significant interactions with long chain *n*-alkanes and fatty acids. More recent anc3–6-CYP52 structures displayed a similar interaction profile with the last ancient CYP52 but expanded their interactions with C11-C14 *n*-alkanes (Fig. 4A). With regards to anc1-CYP52, it only interacted moderately and exclusively with some branched alkanes (Fig. 4B). In contrast, anc2-CYP52 paralogous structures of *Y. lipolytica* exhibited wide interaction abilities and high docked energy values with C6-C20 *n*-alkanes and branched alkanes, although only moderate interaction with C8-C10 fatty acids was estimated (Fig. 4B). Unlike their ancestor anc3-6-CYP52, most of the ancestors to CYP52 groups 3, 4, 5, and 6 showed moderate or high interactions with C10-C20 n-alkanes. Likewise, each of these ancient CYP52 groups interacted highly with C8-C16 fatty acids. This capacity was absent in their anc3-6-CYP52 and last ancient-CYP52 ancestors (Fig. 4B).

### Analysis of primary and tertiary CYP52 structures

The amino acid similarity/identity values of ancient reconstructed and actual CYP52 enzymes are included in Table S1. In general, the similarity/identity among all actual orthologous and paralogous CYP52 sequences was between 48.9–72.5/36–65.5%. Meanwhile, the levels of amino acid similarity/identity among all ancient CYP52 displayed was between 79.9–97.8/63.6–97%. The last ancient CYP52 and anc3-6-CYP52 presented a high percentage of similarity/identity of 97/97.5%.

**Figure 5:**
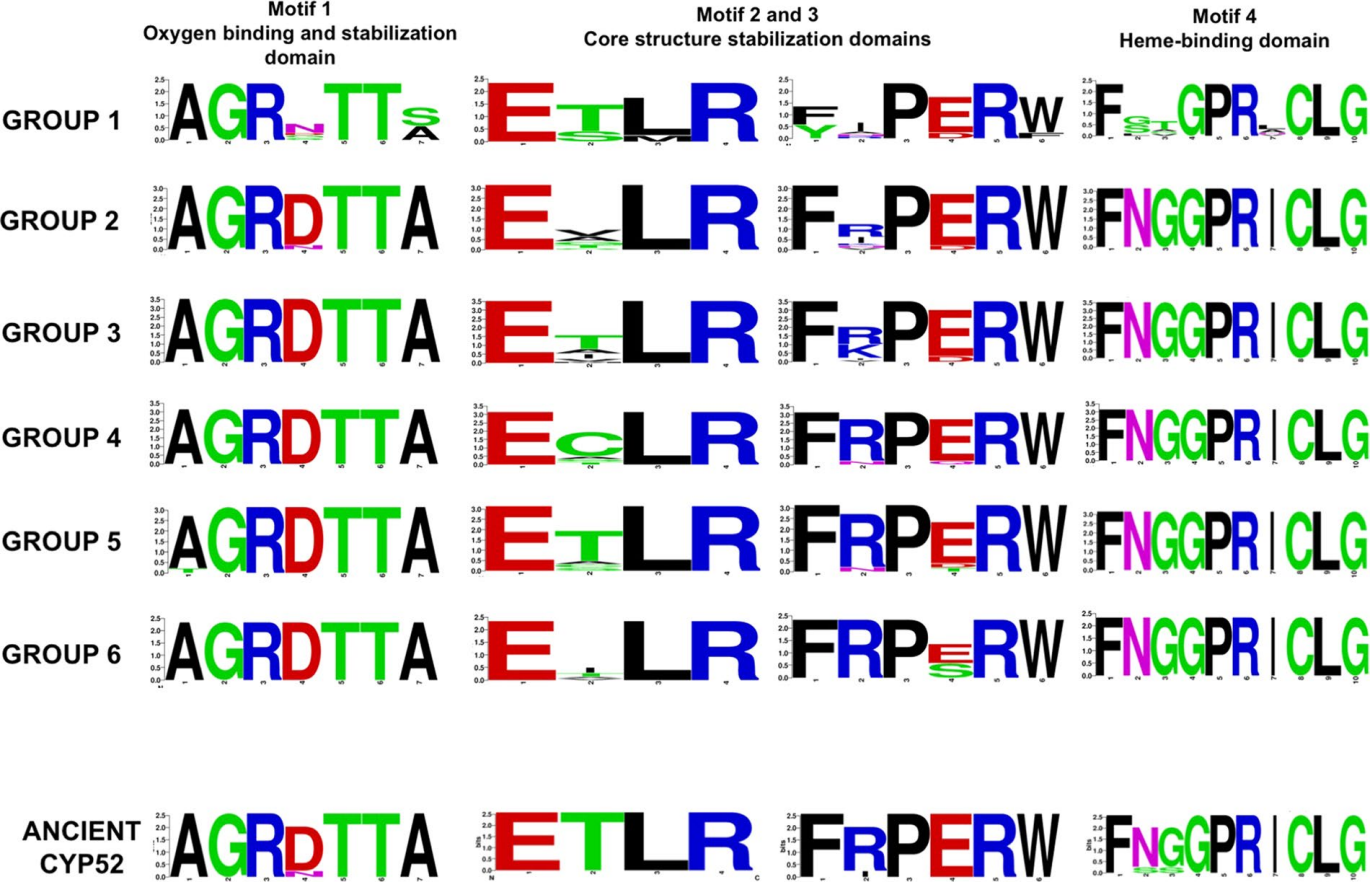
Sequence logos of the conserved motif from modern CYP52 orthologous and paralogous groups compared with motif sequences of ancient CYP52 enzymes. Multiple alignments were performed with MUSCLE v3.8.3 using SeaView 4 v. 4 software, and the consensus logos were generated using WebLogo (http://weblogo.threeplusone.com/create.cgi,).

Motif 1 of CYP52, presented some variability at position 4, with the presence of small and polar amino acids (D, N, and S), but the majority of groups and last ancestral sequence have an aspartic acid residue (Fig. 5). Another change was observed at position 7, where an alanine was replaced by serine only in the orthologous group 1. Motif 2 displayed conservative hydrophobic amino acids at positions 2 and 3, where threonine and leucine, respectively, were the predicted amino acids in the last ancestral CYP52. In motif 3, the CYP52 groups displayed a conservative variability of aromatic amino acids at position 1 (F and Y) and 6 (W and F). Furthermore, there were a variety of polar and mainly negative amino acids observed at position 4 (E, D, S, and T). Except for group 1, motif 4 was highly conserved.

**Figure 6:**
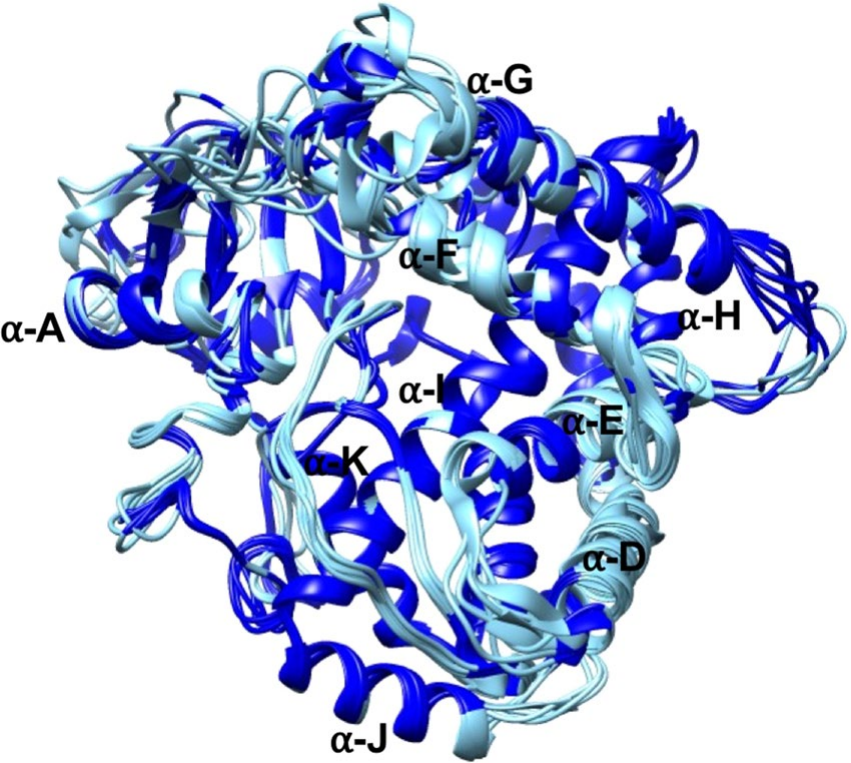
Fold conservation of the ancient CYP52 enzymes. The three-dimensional structures were predicted by Modeller 9.13 using the human CYP46A1 and superimposed using UCSF Chimera software. The conserved regions are highlighted in dark blue, and variable regions are highlighted in sky blue.

Superimposing of the eight three-dimensional structures of ancient CYP52 displayed RMSD values between 0.4 and 1.65 and pairwise levels between 37.47 and 91.32% (Fig. 6; Table S4). The α-A, α-G, and α-J helixes presented moderate conservation. Superimposition of the ancestral CYP52 structures highlighted highly conserved regions. There were also variable regions in the α-F and α-G helices associated with the substrate access channel and the α-D and α-E regions, the latter of which is associated with the catalytic pocket (Fig. 6).

**Figure 7:**
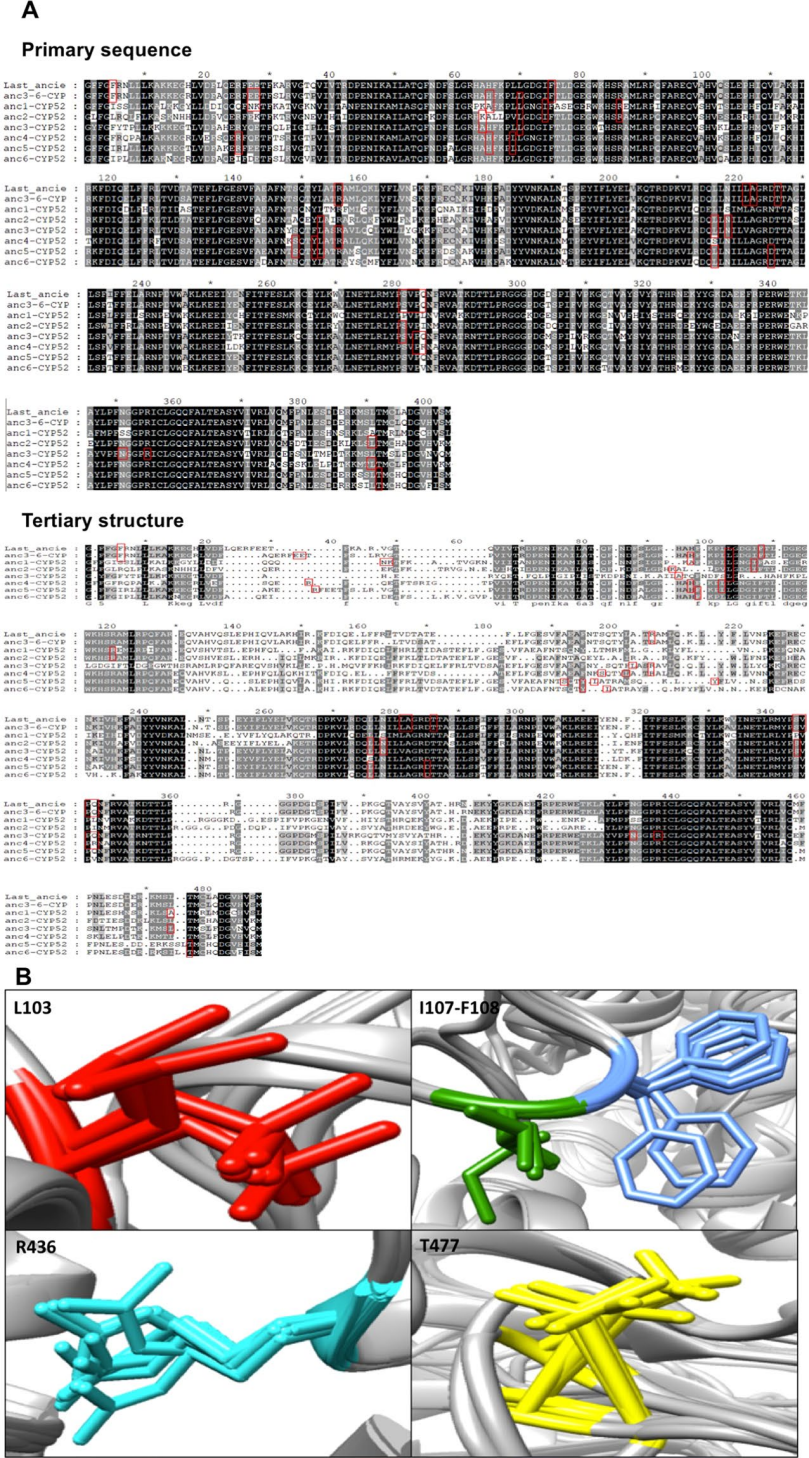
Location and spatial orientation of the amino acid residues of the active site of the ancient CYP52 enzymes. (**A**) The location of the residues was performed in primary structure alignment and tertiary structure alignment. Amino acid residues are highlighted in red boxes. (**B**) Spatial orientation of the conserved amino acid residues of the active site. Amino acid residues are colored in red (L103), green (I107), blue (F108), aquamarine (R436) and yellow (T477).

Alignment with primary sequences of amino acid of the all ancient CYP52 revealed that catalytic pocket amino acids interacted with hydrocarbon or fatty acid ligands in conservative regions (Fig. 7A). However, the alignment with tertiary structures relocated the position of some residues along structure, some of which were distributed in variable regions (Fig. 7B). Although many amino acids remained in conserved regions (e.g., L103, I107, F108, R436, and T477), they still presented differences in their spatial orientation (Fig. 7B).

## Discussion

Cytochrome CYP52 enzymes form a protein family of orthologs and paralogs that are involved in the assimilation of alkanes and fatty acids in Saccharomycetales^7^. CYP52 members are present in Saccharomycetales that comprise CTG clade, whereas CYP52 enzymes are absent in Saccharomycetales of the WGD clade. CYP52 enzymes are widely distributed among other more ancient fungal taxa^17^, and thus it is possible that a gene loss event of ancient CYP52 in early evolution explains the actual absence of CYP52 genes among WGD clade members. The maximum likelihood phylogenetic reconstruction of CYP52 suggests a complex scenario of duplication and depurative events. A total of three pure orthologous groups (1, 5, and 6), two orthologous/paralogous groups (3 and 4), including several species and one paralogous group that exclusively contained *Y. lipolytica*, represent the actual phylogeny. Many putative depurative events were recognized in each orthologous group, but an amazing set of recent paralogous proteins emerged in all groups. No species had a representative CYP52 copy in the five groups of orthologous or orthologous/paralogous. However, the yeast of the Debaryomycetaceae family maintain at least one ortholog in the five groups.

Similar expansion events by gene duplication of acylglicerol lipases, asparaginases, aspartyl proteases, yapsins, DUP240, and even other P450 protein families, among others, have been detected in Saccharomycetales yeast^32–35^. The CYP52 family was expanded and purified as a result of several ancient and recent gene duplication and gene loss events along their evolutionary history. This scenario is common for other protein families^33,36,37^. The actual CYP52 protein distribution can be explained as a consequence of a series of ancestral duplication events. Genome duplication followed by massive reductive evolution is a phenomenon properly documented in Saccharomycetaceae family^21,25,38^. However, gene gains and losses in multi-family protein families are poorly documented. This fact means that it is not easy to establish a relationship between the paralogous gene content of each species with its environment and selection pressures. However, as a general rule, species like *C. lusitaniae* are isolated frequently from carbohydrate-rich and lipid-poor environments^39,40^ and harbor a restricted number of CYP52 paralogs. In contrast, *Y. lipolytica* strains are frequently isolated from carbohydrate-poor and lipid-rich environments, including chesses^41^, oil-contaminated soils ^42^ and fermented foods^43^, and harbor a greater number of CYP52 paralogs.

The case of *C. tropicalis* is very particular because this species has been isolated from natural environments, including hydrocarbon-^44,45^ and heavy-metal-contaminated soils^46,47^ and immunocompromised human infections^48^. *C. tropicalis* ENCB-2409 strain used in this study was isolated from the soil of a gas station contaminated with gasoline and assimilated *in vitro* several hydrocarbons and fatty acids. Three clinical strains of this species were included in the phenotypic analyses of this study, but the RAPD profile and dendrogram revealed substantial genomic differences among some environmental and clinical *C. tropicalis* strains. A comparative genomics study of *C. tropicalis* strains isolated from different environments will be necessary to determine whether this correlation between the genetic content of CYP52 enzymes and the habitat of this versatile yeast species exist, if different ecotypes can grow in different habitats, or even if new species must be defined.

*M. guilliermondii* also requires special discussion. This yeast is isolated from carbohydrate-rich environments such as fruits and juices^49^, soil polluted with hydrocarbons^50^, and immunocompromised human infections^51^. The *M. guilliermondii* ENCB-M used in this work, which was isolated from digestive tract of *Dentroctonus rhizophagus*, a bark beetle endemic to Northwest Mexico^52^, displayed a wide assimilation spectrum of alkanes. The genus *Dendroctonus* occupies an ecological niche where alkanes, for example, *n*-heptane, are present in moderate concentrations^53^. Perhaps the capability of *M. guilliermondii* ENCB-M is associated with the constant exposition the gut microbiota of *Dendroctonus rhizophagus* to volatile alkanes. However, the presence of a reduced number of CYP52 paralogs in *M. guilliermonii* represents a particular phenomenon where the number of CYP52 cytochromes and the number of hydrophobic substrates that this yeast can utilize are not correlated. Similar to *M. guilliermondii*, *K. ohmeri* displays a broad hydrocarbonoclastic phenotype, but its CYP52 content is very reduced^20^. Therefore, in *M. guilliermondii* and other yeasts with few CYP52 enzymes, CYP52 duplication was apparently not necessary in their adaptation to hydrocarbon-rich environments. Consequently, another phenomenon of adaptation could be involved in the optimization of their hydrocarbonoclastic capability.

*C. tenuis*, *D. hansenii*, and *C. stipitis* are isolated mainly from fermented foods and beverages, gut insects, and plants; they harbor a moderate or low number of CYP52 orthologs. These species are not commonly isolated from lipid- or hydrocarbon-rich environments, a finding that reflects their moderate or poor hydrocarbonoclastic phenotype. However, these species possess a versatile ability to assimilate carbohydrates but not hexadecane^54^. The presence of CYP52 enzymes in species from hydrocarbon-poor environments is possibly required to degrade fatty acids from host or fermentation culture by ω−oxidation. Additionally, other yeast species from environments that lack hydrocarbons, including *Candida californica*, *Candida zeylanoides*, and *Y. mexicana*, were included in this work. They were capable of degrading a limited number of *n*-alkanes, but CYP52 paralogs were not analyzed, because the genome project or the CYP52 sequences of these yeast in databases are not available yet. These results suggest the hydrocarbonoclastic traits are strongly conserved in most of Saccharomycetales.

In *C. parapsilosis*, *C. tropicalis*, and *Y. lipolytica*, all of which harbored the greatest number of CYP52 proteins, the extensive expansion of this family could be associated with an evolutionary process for assimilating long chain *n*-alkanes and fatty acids under several environmental and nutritional conditions. Similar phenomena were observed in other expanded paralogous protein families, including hexose transporters^55^, antifreeze glycoproteins^56^, histones^57^, lipases^58^, and ABC transporters involved in drug resistance^59^, among others. The phenotypic plasticity derived from differential expression of an expanded paralogous family is valuable under changing environmental conditions^60,61^. The expansion of CYP52 paralogs is probably a consequence of the molecular complexity of hydrophobic substrates and variable physicochemical and chemical conditions of the habitat occupied by these yeasts. The extension of the ecological niche occupied by these hydrocarbonoclastic yeasts would be directly proportional to the versatility to use a great diversity of hydrophobic compounds as main carbon source under different environmental conditions^62^.

Yeasts from the WGD group, including *K. lactis*, *C. piceae*, *C. americana*, and *C. bracarensis*, can assimilate C8-C16 *n*-alkanes. No available genome projects exist for *C. piceae* and *C. americana*. However, although *K. lactis* and *C. bracarensis* genome projects were explored, no homologous CYP52 enzymes were detected by BLAST and Hidden Markov Models. In the Fungal Cytochrome P450 Database, no CYP52 enzymes were recognized in the *K. lactis* genome (http://p450.riceblast.snu.ac.kr/). Obviously, the genes for degradation of alkanes in *K. lactis* and *C. bracarensis* have yet to be recognized and possibly belong to a new family of oxidizing enzymes.

Although the expansion of the CYP52 enzymes plays an important role in the adaptation to hydrophobic-rich environments in Saccharomycetales, most of CYP52 paralogs in a species exhibit partially overlapping affinity for hydrophobic substrates. However, other sets of CYP52 enzymes exhibit a clear substrate specialization for fatty acids or long-chain alkanes, as can be observed in *Y. lipolytica*^18,19^. In general, the functional specialization of the P450 superfamily members is recognized as low, a designation that assumes a certain functional redundance^63,64^. These results could be supported by docking analysis of ancient CYP52 performed in this work, which also revealed moderate to low substrate redundancy among them. However, in our opinion, this statement was hastily made because it was based only on results of semi-quantitative expression or phenotype in *CYP52* null mutants. Both studies were conducted with few variations in environmental conditions, a design that severely limited any difference in differential expression and therefore in the definition of the function.

Docking simulations in ancient CYP52 suggest that this protein family had as primordial function to use C4-C10 short-chain alkanes, and the interactions with long-chain alkanes and fatty acids could be a derived function. Recent enzymes appear to have loosened and/or broadened the ancient phenotype of assimilation of short chain alkanes, as demonstrated by docking and alkane assimilation test. Unlike some yeast enzyme families, including α-glucosidases derived from ancient enzymes with functional promiscuity, modern α-glucosidases display clear signals of subfunctionalization and catalytic optimization^65^. According to hypothesis of the functional evolution of the enzymes^66,67^, CYP52 suffered a functional innovation event. However, because docked energy values among ancient CYP52 are similar, the functional optimization is not yet evident. Furthermore, in the ancestor CYP52 paralogous families, there was a promiscuous interaction of the enzyme with hydrophobic substrates. However, there is obvious partial specialization by the type of substrate in the actual CYP52 paralogous families. This specialization process is possibly still ongoing due to the recent duplication events that gave origin to this family of paralogous.

Actual and ancient CYP52 orthologs and paralogs present among them moderate amino acid similarity, but there is a high conservation of relevant motif sites of CYP52 enzymes. Similarly, motif sites for other fungal P450 proteins revealed conserved modifications, namely in EXXR and FXXGXRXCXG motifs^68^. However, there are important differences in the catalytic pocket, substrate entrance channel, and three-dimensional structure of CYP52 of Saccharomycetales. Currently, the role that these variations play in the levels of CYP52 family redundancy and specialization are unclear. All ancient CYP52 paralogous share a moderate three-dimensional structure fold, a phenomenon previously observed in the structure of other bacterial and mammalian P450s^69^. Similarly, ancient α-glucosidases^65^ and β-lactamases^70^ maintain conserved tertiary structures, but fixed mutations that affect the catalytic pockets. No significant mutations were detected in the catalytic pocket. However, the spatial orientation of the amino acid residues in the active site could explain the changes in the interaction of ancient CYP52 with different alkanes and fatty acids.

Defining the function of paralogous genes is not a trivial objective. For this reason, we propose that, before concluding whether a paralogous family has functional redundancy, the following aspects should be explored: phylogeny, transcriptional differential expression, enzyme-substrate docking, comparative protein structural analysis, phenotypic profile of substrates assimilation, heterologous expression, and biochemical characterization. The extended family of CYP52 protein paralogs and orthologs is likely evolving toward functional specialization. Certain redundancy for substrates and sharing transcriptional expression profiles in adaptation to physical, physicochemical, and chemical conditions of a complex ambient will probably be maintained.

## Material and Methods

### Phylogenetic analysis of CYP52 family

A total 77 non-redundant amino acid sequences of orthologous and paralogous CYP52 proteins were collected from 14 yeast annotated genome projects from the public NCBI (https://www.ncbi.nlm.nih.gov/) and KEGG (www.genome.jp/kegg/) databases. CYP52 amino acid sequences from *C. maltosa* were obtained from GenBank^13^. Sequences with an expected value <1e-5 and subject coverage> 95% were selected for the analysis. A multiple alignment of sequences was performed with MUSCLE v. 3.8.31, included in the program SeaView version 4.7^71,72^. Selection of the best evolutionary model test was performed using the web server ProtTest 3^73^. The WAG + F + I + G model test was selected for the phylogenetic analysis, using the -lnL, AIC and AICc selection criteria (-lnL= 49889.9, AIC = 100092.04, AICc = 1.00). A maximum-likelihood phylogenetic tree was constructed with MEGA X, supported with a bootstrap analysis of 1,000 replicates^74^. Internal nodes with a low supported Bootstrap (<0.5) were collapsed. The phylogenetic tree was edited with the web server iTOL^75^.

### Phylogenomics analysis with Virtual Analysis Method for Phylogenomic fingerPrint Estimation (VAMPhyRE)

The phylogenomic tree was constructed with 13 complete and draft fungal genomes by identifying the virtual genome fingerprints (VGFs) for each genome, using VAMPhyRE (Mendez-Tenorio *et al*. manuscript in preparation). First, a single string of concatenated contigs or chromosome sequences for each genome was built. Next, a virtual hybridization analysis was made with a collection of 15,264 highly diverse VAMPhyRE probe set 13-mers (VPS-13) not allowing any mismatch; both sense and antisense strands were analyzed. The analysis yields a collection of hybridization sites, which constitutes a Virtual Genomic Fingerprint (VGF). Genomic distances were estimated by calculating the number of homologous sites shared among pairs of genomic VGFs. This is accomplished by applying an extended-match strategy of five bases to the left and right of the genomic alignment of each shared site and a threshold of 21 bases to eliminate non-homologous sites. Those extension and threshold values were properly stablished through a previous optimization method involving the comparison of both related and non-related sequences. From the fraction of homologous shared sites, between pairs of VGFs a distance value was determined according to Nei and Li^76^ to calculate a matrix of distances. The distance matrix was used to build the phylogenomic tree using MEGA X^74^. The edition and annotation of the tree were similarly made with this program.

### RAPD polymerase chain reaction (RAPD-PCR) of environmental and clinical *C. tropicalis* strains

RAPD-PCR was performed using the primers OPE4 (5′-AGCTGACCGT-3′), OPE18 (5′-GGACTGCAGA-3′), and OPA18 (5′-GTGACATGCC-3′)^77^. The PCR reaction and thermocycler parameters were based on a previous protocol^78^. The generated amplicons were separated in a 2% agarose gel electrophoresed at 80 V for 120 min. A combined presence (1)/absence (0) binary data matrix was generated from the obtained band pattern. The matrix was used for the construction of a dendrogram using NTSYSpe 2.02 software^79^ using the unweighted pair group method and arithmetic mean (UPGMA) and the Jaccard coefficient. The confidence of the nodes of the dendrogram was evaluated with the Jackknife method, with a total of 1,000 replicates.

### Hydrocarbon and fatty acid assimilation assays

The assays were performed in liquid mineral medium in 96-well plates according to a previously described protocol^20^. In brief, the C4-C10 *n*-alkanes and branched alkanes were tested in the vapor phase, while fatty acids and *n*-hexadecane were added to the liquid phase at a 1% final concentration. The plates were incubated at 28 °C for 10 days. The yeast growth was estimated by absorbance at 620 nm with a Multiskan™ FC Microplate Photometer (Thermo Fisher Scientific®). The assays were performed in triplicate and analyzed as parametric data, fulfilling normality test. Multiple comparisons were two-tailed and evaluated with a Two-way analysis of variance (ANOVA) followed by Tukey’s post-hoc test with GraphPad Prism 6 to determine significant differences. The information of the assimilation carbohydrate profiles of the yeast species was obtained from previously published data^54^.

### Ancestral sequence reconstruction

The ancestral reconstruction of the ancient CYP52 enzymes for each orthologous group was inferred using PAML package version 4.8, which was computed with LG, WAG, and JTT as evolutionary models^80^. The analysis and evaluation of the obtained ancient sequences was performed according to a previously described methodology^65^. The codeml script included in the PAML package was employed using the default parameters. Both marginal and joint reconstruction methods were computed in the inference of ancestral proteins. Sequences obtained by marginal reconstruction under the JTT model were selected for three-dimensional modelling of ancient CYP52 proteins. To infer the most ancient ancestors of the yeast CYP52, a total of six ancient sequences were obtained.

### Modelling of three-dimensional ancient CYP52

The hypothetical three-dimensional structures of ancient CYP52 were obtained using the threading homology method^81^. Searching for templates required for the threading modelling method was performed using the web server PGenThreader (http://bioinf.cs.ucl.ac.uk/psipred). The modelling was performed with Modeller 9.13 using the crystal structure of human CYP46 A1 cytochrome (PDB entry: 3MDM) as template^82^. The structures with the lowest objective function values were visualized using UCSF Chimera^83^. The evaluation of the structures by stereo-chemical restriction determinations employing Ramachandran plots and RMSD were performed with PROCHECK v.3.5.4^84^ and Dali server (http://ekhidna2.biocenter.helsinki.fi/dali/), respectively.

The three-dimensional structures were subjected to molecular dynamic simulations. The parameters for simulations were established with VMD^85^. The simulation was performed using NAMD2 software^86^, using the force fields from CHARMM36m^87^ and including all Langevin dynamic parameters and a time step of 2 ft/step. A rigid cell was used during the process. The simulation was performed in 2,000,000 steps for a total time of 1 ns, using a water box with a size of 10 Å embedded on 0.15 mM NaCl. The results of the trajectory and RMSD of each step of the simulation were visualized and graphed in VMD.

### Docking of alkanes and fatty acids with ancestral CYP52 structures

The structures of 32 *n*-alkanes, branched alkanes, and saturated and unsaturated fatty acids were selected and downloaded from the free ZINC12 database (http://zinc.docking.org). The representative optimized three-dimensional ancient CYP52 optimized by molecular dynamics were selected for docking test. AUTODOCK version 4.0 was employed to evaluate binding orientations with the optimized structures of deduced ancient CYP52 previously obtained by molecular dynamic^88^. The graphical interphase AutoDockTools was used to determine the docking parameters. Ancient CYP52 enzymes were prepared by adding polar hydrogens, merging non-polar hydrogens, and removing water molecules and ions. Grid maps were computed by estimating the Gasteiger-Marsili partial charges and Lennard-Jones parameters 12–10 and 12-6 and electrostatic potential parameters. The grid dimension was 126 ×126 ×126 Å^3^, with points separated by 0.375 Å. Random starting positions, orientations and torsion were established for all ligands. Default values of translation, quaternation, and torsion steps were used for the simulation. The Lamarckian genetic algorithm was applied for minimization, using default parameters by pseudo-Solis and Wets method. Guided molecular dockings were executed with a total of 100 runs. Docking results were analyzed in AutoDockTools and edited in Discovery 4.0 Client (https://www.3dsbiovia.com/).

### Amino acid sequence and structural analysis

An alignment of the ancient CYP52 with their respective actual paralogous CYP52 was performed with MUSCLE v3.8.31^71^. Sequence modification at the motifs sites was analyzed in the alignment and visualized with the web server WebLogo (http://weblogo.berkeley.edu/logo.cgi). The pairwise levels of similarity/identity were computed with MatGAT v2.02 software^89^. The superimposition, determination of pairwise levels of similarity and RMSD of the three-dimensional structures of ancient CYP52 were performed with MatchMaker and Match -> Align programs included in UCSF Chimera software^83^.

## Supplementary information

Supplemental information.

Supplemental information 2.

Supplemental information 3.

Supplemental information 4.

Supplemental information 5.

Supplemental information 6.
