# Supplementary figures and images for: Phylogeny, evolution, and potential ecological relationship of cytochrome CYP52 enzymes in Saccharomycetales yeasts
Source: Sci Rep. 2020 Jun 24;10:10269. doi: 10.1038/s41598-020-67200-5 (PMC7314818; doi:10.1038/s41598-020-67200-5)

**A****OPE-4**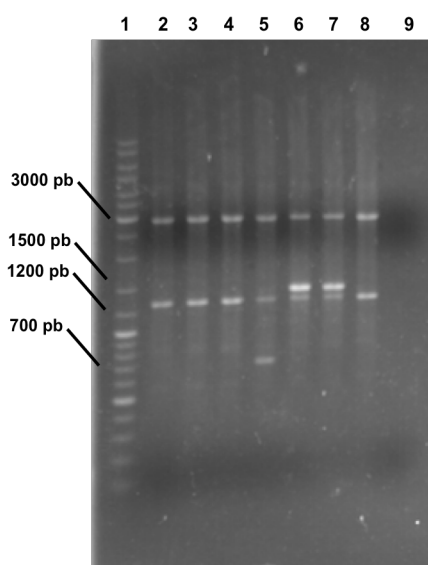**OPE-18**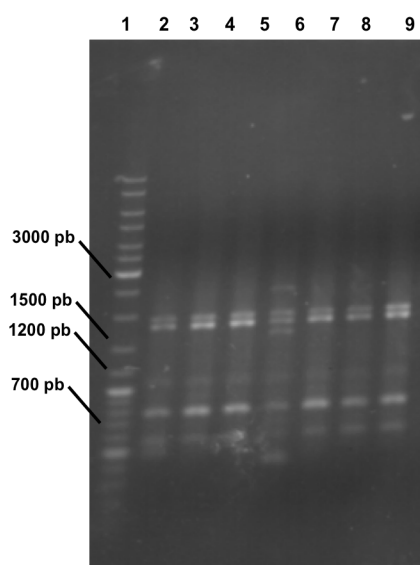**OPA-18**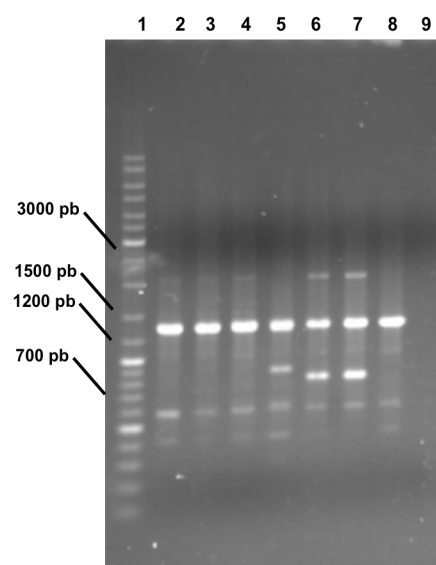**B**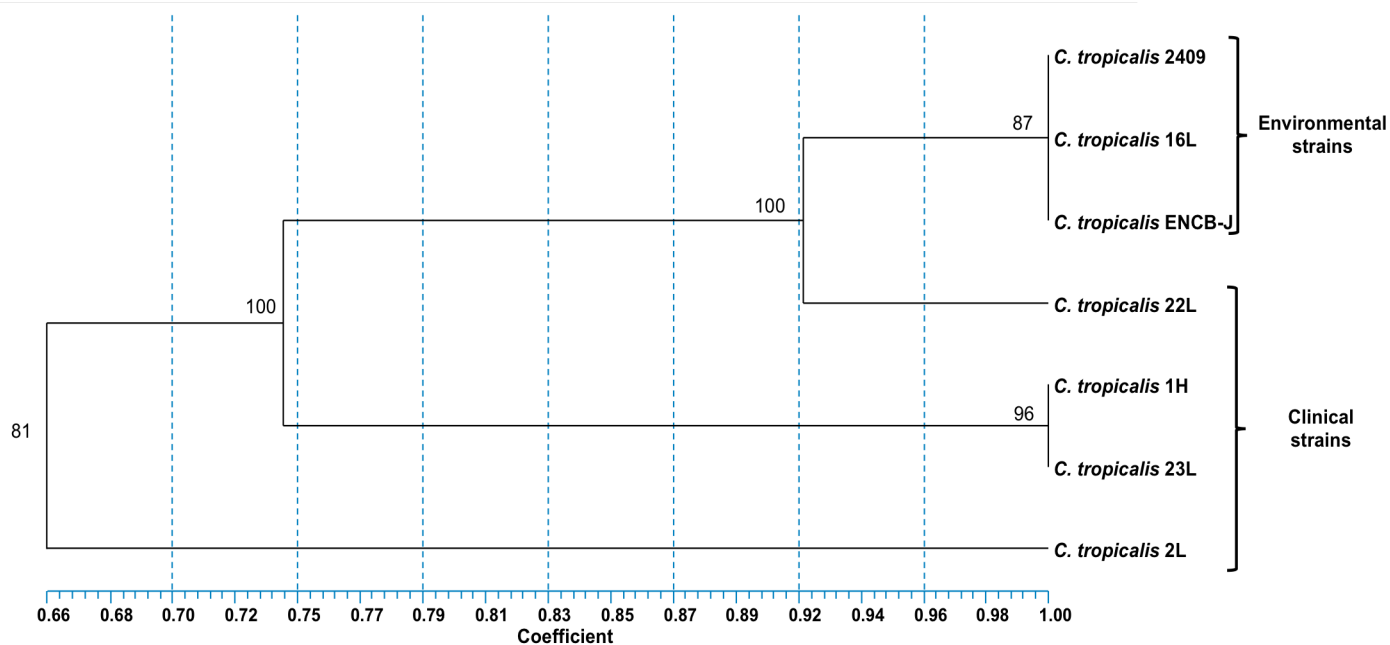

Supplement: Supplementary file 2 — Supplemental information 2. [file 41598_2020_67200_MOESM2_ESM.pdf]

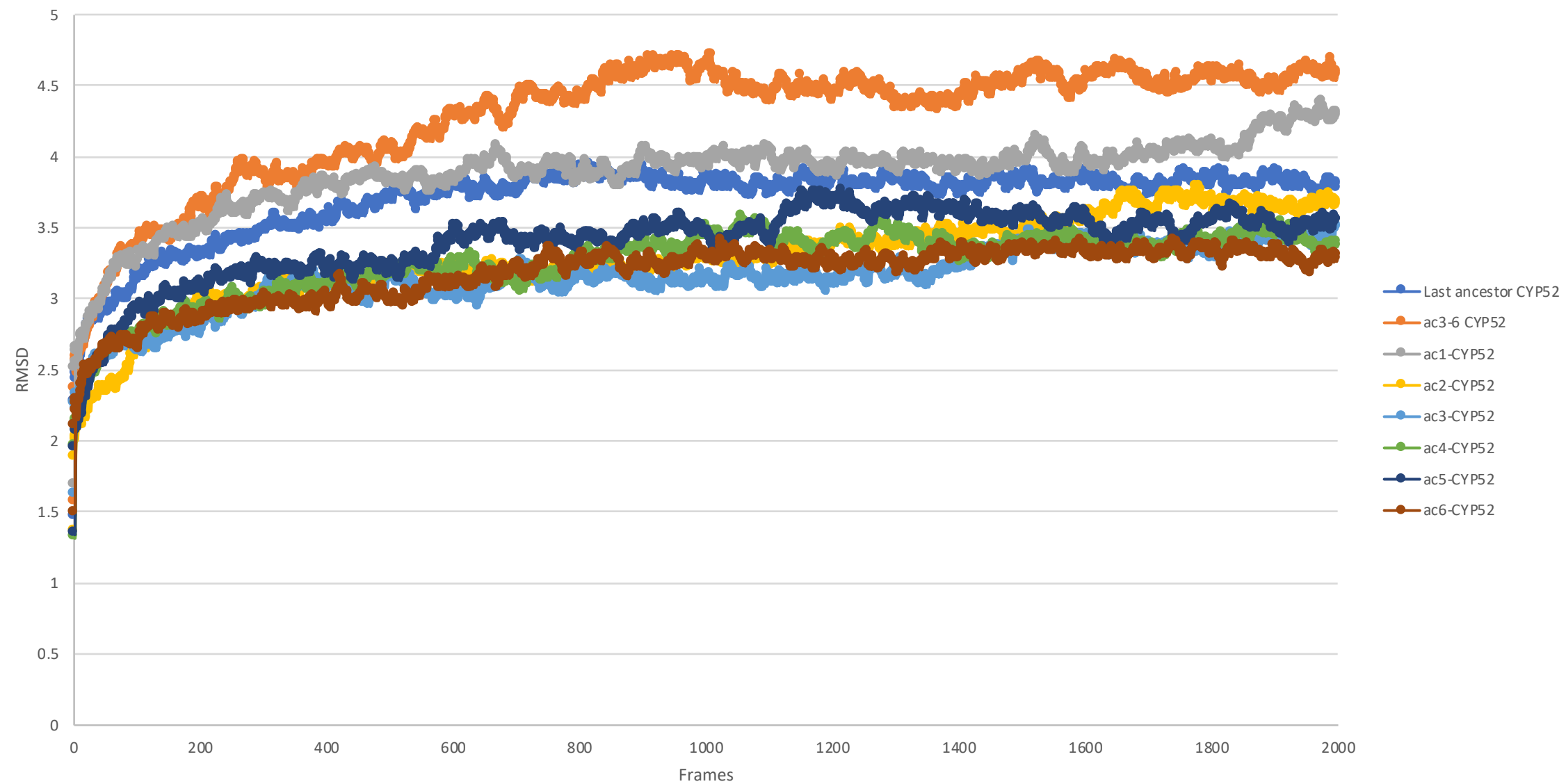

Supplement: Supplementary file 3 — Supplemental information 3. [file 41598_2020_67200_MOESM3_ESM.pdf]
